# The Berkeley PET Imaging Pipeline: Harmonization of Alzheimer’s disease PET biomarkers across studies

**DOI:** 10.1002/alz.095834

**Published:** 2025-01-09

**Authors:** Theresa M. Harrison, Susan M. Landau, Suzanne L. Baker, Martin Boswell, William J. Jagust

**Affiliations:** ^1^ University of California, Berkeley, Berkeley, CA USA; ^2^ Lawrence Berkeley National Laboratory, Berkeley, CA USA

## Abstract

**Background:**

Collection of neuroimaging data is resource‐intensive. Multi‐site studies have emerged as an effective way to amass large collections of PET scans, but data collected across many sites and scanners require specialized processing approaches. The Berkeley PET Imaging Pipeline (BPIP) and tools for numerical data post‐processing allow us to 1) process multi‐site and multi‐study PET scans using harmonized, largely automated tools and 2) disseminate numerical quantitative data.

**Method:**

PET data are harmonized by first using standard, tracer‐specific PET protocols and applying spatial smoothing to achieve a uniform resolution across scanners and sites. Using BPIP we complete MRI‐dependent or MRI‐free processing of PET scans which includes 1) definition of ROIs (MRI‐dependent: FreeSurfer Desikan‐Killiany in native space; MRI‐free: normalized probability Desikan‐Killiany in template space), 2) intensity normalization using a primary reference region, and 3) calculation of standardized uptake value ratios (SUVRs) across atlas ROIs, composite ROIs (e.g., temporal metaROI for tau tracers) and alternative reference regions (e.g., for longitudinal analyses). Tau‐PET scans are also partial volume corrected. BPIP runs on the XNAT platform on a dedicated server. For the MRI‐dependent pipeline, visual quality control (QC) is performed on automatically generated QC images that allow us to assess co‐registration and ROI delineation. We use a suite of software tools to curate and format the standardized numerical data. For studies that allow it, we release numerical quantitative PET data to the research community. For research conducted in our lab, we create datasets that combine neuroimaging data with other variables (e.g., demographic, genetic, cognitive).

**Result:**

Using our current approach, we have processed over 20,000 PET scans across 8 Ab and tau tracers and corresponding T1 MRIs from more than a dozen studies. We have made data from more than 10,000 Ab and tau‐PET scans available in publicly shared ADNI, SCAN and POINTER Imaging datasets. For studies where we are not able to share numerical data publicly, we share data with individual investigators when formal approval is granted by the original study.

**Conclusion:**

BPIP and associated software for data post‐processing are a powerful engine supporting the analysis and dissemination of Alzheimer’s disease PET biomarker data.

